# ASO Author Reflections: Socioeconomic Disparities in Breast Tumors Persist Despite Universal Healthcare

**DOI:** 10.1245/s10434-026-19768-x

**Published:** 2026-05-04

**Authors:** Carl Sars, Ebba K. Lindqvist

**Affiliations:** 1 Department of Molecular Medicine and Surgery, Karolinska Institutet, Stockholm, Sweden; 2 Department of Clinical Science and Education, Stockholm South General Hospital, Karolinska Institutet, Stockholm, Sweden; 3 Department of Surgery, Stockholm South General Hospital, Stockholm, Sweden

## Past

Sarcomas of the breast are rare, and heterogeneous tumors encompassing phyllodes tumors, angiosarcomas, and soft-tissue sarcomas collectively represent less than 1% of all breast malignancies. Surgery remains the cornerstone of treatment, yet factors influencing long-term survival remain poorly understood. Socioeconomic status (SES) is a well-established determinant of cancer outcomes in common malignancies such as breast carcinoma,^1^ but its role in rare breast tumors had not been studied using individual-level data. Existing studies relied on neighborhood-level socioeconomic indices^2^ or insurance status as proxies,^3^ limiting their interpretability and generalizability. A particular knowledge gap existed regarding whether SES-related disparities persist within universal healthcare systems, where formal access to treatment is equal for all residents. In the present study, we sought to investigate the association of SES, defined as highest level of attained education and household disposable income, and overall survival among women with sarcomas of the breast in Sweden.^4^

## Present

In this nationwide, population-based cohort study of 473 women diagnosed with sarcomas of the breast in Sweden between 1993 and 2018, we found that lower household income, but not educational attainment, was associated with worse overall survival, even after adjustment for age and comorbidity.^4^ Women in the lowest income quintile faced more than twice the mortality risk compared with those in the highest quintile. This association was most pronounced among patients with borderline phyllodes tumors. Our findings demonstrate that survival disparities persist even within a system designed to provide equal access to care, suggesting that low income reflects dimensions of disadvantage that extend beyond formal access to care, and that targeted interventions beyond healthcare provision may be needed to address these disparities.

## Future

Future studies should continue to examine the association between tumors and long-term outcomes. In the setting of sarcomas and phyllodes tumors of the breast, attention should be directed at exploring disease-specific survival as a complementary endpoint, requiring clinical case-by-case review to minimize misclassification in registry-based cause-of-death data. Distinguishing primary from secondary angiosarcomas, the latter arising after radiation therapy, would further refine prognostic analyses within this heterogeneous group. Larger international collaborations are needed to achieve sufficient statistical power for subgroup analyses across individual tumor categories. Most importantly, the mechanisms underlying income-related disparities—whether driven by differences in health literacy, social support, treatment adherence, or clinician implicit bias—warrant targeted investigation to inform policy interventions that can meaningfully reduce inequities in survival for patients with these rare tumors.
